# Clinical Evidence and Potential Mechanisms in Treating Radiation Enteritis with Modified Baitouweng Decoction

**DOI:** 10.1155/2023/9731315

**Published:** 2023-01-30

**Authors:** Zihong Wu, Bei Yin, Ziming Wang, Enfeng Song, Fengming You

**Affiliations:** ^1^Hospital of Chengdu University of Traditional Chinese Medicine, Chengdu, China; ^2^School of Second Clinical Medicine, Guangzhou University of Chinese Medicine, Guangzhou, China; ^3^Department of Traditional Chinese Medicine, Renmin Hospital of Wuhan University, Wuhan, China

## Abstract

**Objectives:**

To perform a meta-analysis and network analysis identification to evaluate the efficacy, safety, and potential mechanisms of modified Baitouweng decoction (mBTWD) in the treatment of radiation enteritis.

**Methods:**

We searched PubMed, Embase, Cochrane Library, Web of Science, CNKI, Wanfang Databases, SionMed, and Chinese Scientific Journals Database to collect the randomized controlled trials (RCTs) of mBTWD treating radiation enteritis. Rev.Man 5.3 and Stata 14.0 software are employed for meta-analysis. The GRADE online tool is used to evaluate the quality of evidence. Network analysis and molecular docking approach are applied to predict the potential targets and ingredients of representative drugs in mBTWD for the treatment of radiation enteritis.

**Results:**

Seventeen studies are eventually included, covering a total of 1611 patients: (1) The clinical efficacy is significantly higher in mBTWD groups than in control groups (RR = 1.24, 95% CI (1.17, 1.32), *P* < 0.00001). (2) mBTWD has certain advantages in improving TCM syndromes (MD = −3.41, *P* < 0.00001). (3) mBTWD has a certain positive effect on the improvement of intestinal signs and symptoms (RR = 1.23, *P*=0.0001; OR = 3.51, *P* < 0.00001). (4) Indexes including CRP, KPS, and OB, are better in mBTWD groups than in control groups (*P* < 0.00001, *P*=0.002, *P*=0.03), but the credibility is downgraded for a small sample size. Adverse events and recurrence rates require further confirmation with larger sample sizes. (5) Univariate meta-regression for clinical efficacy shows none of the coefficients are significantly associated with the estimated risk ratio. The clinical efficacy overestimates about 4.9% from publication bias. The quality of the included studies is low according to GRADE evidence. (6) Quercetin, isorhamnetin, and beta-sitosterol are the main ingredients from representative drugs in mBTWD and its key targets are MYC, TP53, and MAPK14/MAPK1.

**Conclusions:**

mBTWD may be effective in the treatment of radiation enteritis, but its long-term benefits, safety, and molecular mechanisms remain unclear due to the poor quality of the evidence. Larger sample sizes, high-quality studies, and basic research are essential in the future.

## 1. Introduction

Radiation therapy is an important treatment therapy for pelvic/abdominal malignancies such as cervical cancer and rectal cancer. While effectively killing cancer cells and controlling local diffusion and distant metastasis of lesions, radiation enteritis often occurs. Oxidative stress caused by the local high doses of radiation can produce a large number of reactive oxide species (ROS), which can cause DNA damage of normal intestinal epithelial cells, atrophy and ulceration of intestinal mucosa, and finally form radioactive enteritis [1]. Approximately 90% of patients with pelvic and abdominal tumors have been reported to have irreversible changes in their defecation habits after radiation therapy, leading to a decrease in the quality of life of approximately 50% of patients [2]. Patients with radiation enteritis mainly present with diarrhea, bellyache, mucous stool, tenesmus, and anal distention, even serious conditions such as intestinal obstruction, intestinal perforation, and fistula formation. These intestinal symptoms lead to a significant reduction in the patient's willingness to continue with radiation therapy [3, 4]. With the improvement of treatment methods, more and more tumor patients benefit from longer survival due to active management of side effects. The treatment of radiation enteritis is mainly symptomatic treatment such as antidiarrheal, anti-inflammatory, analgesic, and fluid rehydration. Commonly used drugs include dexamethasone, gentamicin, lidocaine, and montmorillonite powder. Moreover, the administration is mostly by reserved enema (Re) or per os (Po). These treatments can alleviate symptoms to some extent, but there is often a risk of incomplete treatment, aggravating infection, and high recurrence rate.

**Figure 1:**
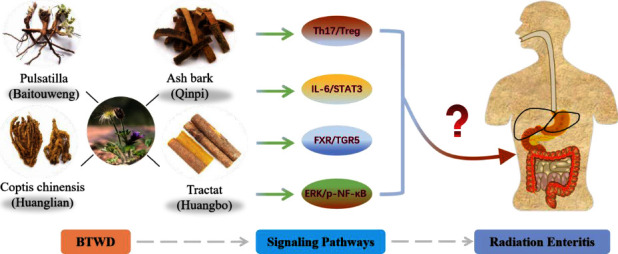
Molecular mechanisms of representative drugs in mBTWD for the treatment of radiation enteritis.

Chinese herbal formula is a long-established, complementary, and alternative medical treatment with curative efficacy, and it is now widely accepted and studied in countries around the world [5]. In recent years, traditional Chinese medicine (TCM) made some achievements in the treatment of radiation enteritis, especially the herbal retention enema reflects the unique advantages of TCM. Baitouweng decoction (BTWD) is from the Treatise on Typhoid Fever, written by Zhang Zhong Jing in the Han Dynasty, and it is a representative formula for the treatment of febrile dysentery. The base ingredients of the modified Baitouweng decoction (mBTWD) are mainly Baitouweng decoction, which consists of four herbs: *Pulsatilla* (Baitouweng), *Coptis chinensis* (Huanglian), tractat (Huangbo), and ash bark (Qinpi). Modern pharmacological studies show that the representative drugs in mBTWD have a good effect on intestinal inflammation. These ingredients regulate intestinal microbiota through FXR/TGR5 and IL-6/STAT3 signaling pathways, restore Th17/Treg cell balance, and improve intestinal immune function and epithelial barrier in mice with enteritis [6–8] (Figure 1). However, the effects and benefits of mBTWD on radiation enteritis are still uncertain, and the mechanism of mBTWD in the treatment of radiation enteritis remains to be further discovered.

In this study, we conducted a meta-analysis to summarize the clinical evidence for mBTWD in the treatment of radiation enteritis. Based on the results of the meta-analysis, we employ network analysis and molecular docking techniques to preliminarily predict the active components and key targets of mBTWD at the molecular level for the treatment of radiation enteritis. This study aims to provide a scientific reference for the clinical application of mBTWD in the treatment of radiation enteritis.

## 2. Materials and Methods

This study is registered with the International Platform of Registered Systematic Review and Meta-analysis Protocols (INPLASY) (https://inplasy.com) as INPLASY 202190053.

### 2.1. Search Strategies

We searched PubMed, Embase, Cochrane Library, Web of Science, China National Knowledge Infrastructure (CNKI), Wanfang Databases, SionMed, and the Chinese Scientific Journal Database from inception until March 1, 2022. The search strategy in PubMed is as follows: ((((“Baitouweng Decoction”(Title/Abstract)) OR (“BTWD”(Title/Abstract))) OR (“Baitouweng Tang”(Title/Abstract))) OR (Baitouweng^*∗*^(Title/Abstract))) AND (((“Radiation enteritis”(Title/Abstract)) OR (“Radiation proctitis”(Title/Abstract))) OR (“Radiation colitis”(Title/Abstract))).

### 2.2. Inclusion/Exclusion Criteria

The inclusion criteria are constructed following the principle of PICOS. (1) Participants: all subjects are patients with abdominal/pelvic tumors complicated with radiation enteritis, regardless of age, sex or nationality. The diagnostic criteria refer to the consensus of Chinese experts on the diagnosis and treatment of radiation enteritis in 2021 [9]. (2) Interventions: the experimental groups are treated with mBTWD alone or combined with conventional western medicine (CWM), and the CWM is the same as the control groups. There are no special restrictions on the dose of herbs, and the methods of administration include retention enema and per os. The treatment course is longer than 14 days. (3) Controls: control groups receive the CWM recommended by expert consensus or just a placebo, and the methods of administration also include retention enema and per os. For example, montmorillonite powder retention enema or oral, or montmorillonite combined with gentamicin, dexamethasone, and procaine for retention enema. (4) Outcomes: the outcome indices mainly include clinical efficacy, TCM symptom score, colonoscopy score/grade, and radiation enteritis grading, KPS score, serum inflammatory factor level, fecal occult blood, adverse events, and recurrence rate. (5) Types of studies: all studies are RCTs and the studies included are not restricted by journals.

Exclusion criteria: (1) Studies on animal testing and basic research. (2) Nonrandomized controlled trial. (3) Studies with data loss, duplicate publications, or unclear outcome indicators. (4) The experimental groups combine acupuncture, massage, and other TCM therapy. (5) The control groups are treated with TCM.

### 2.3. Data Extraction

Two researchers independently conducted the literature retrieval according to the inclusion/exclusion criteria, then performed a preliminary screening by reading titles and abstracts. Next, they read the full text for rescreening and discuss the inconsistencies in screening results. If no agreement is reached, the third researcher will coordinate to solve it. The extracted contents mainly include the following: (1) basic characteristics, including the first author, publication year, sample size, and mean age. (2) Diagnostic criteria. (3) Intervention measures, including drug composition and dose, route of administration, and treatment cycle. (4) Outcome indicators, including efficacy standards and symptom rating scales. (5) Study design and methods. If some details are not fully reported in the study, the researcher will contact the original author.

### 2.4. Quality Evaluation

The Cochrane Handbook [10] is utilized to assess the risk of bias of all included RCTs. And the main types of bias are divided into six categories. (1) Selection bias: random sequence generation and allocation concealment. (2) Performance bias: blindness of participants and personnel. (3) Detection bias: blinding of outcome assessment. (4) Attrition bias: incomplete outcome data. (5) Reporting bias: selective reporting. (6) Other bias: each bias risk is divided into three grades: high bias risk, low bias risk, and unknown bias risk.

### 2.5. Statistical Analysis and GRADE Evidence

RevMan5.3 and Stata 14.0 software is used for data processing. The risk ratio (RR) or odds ratio (OR) are used for dichotomous variables and the weighted mean differences are used for continuous variables. And 95% confidence intervals (CI) are calculated for all effect sizes. Cochran's *Q* and *χ*^2^ test statistics are utilized to test the heterogeneity across studies. The fixed-effects model is adopted with low heterogeneity (*P* > 0.1, *I*^2^ < 50%). If there is heterogeneity in the studies (*P* ≤ 0.1, *I*^2^ ≥ 50%), the random-effects model is chosen. Sensitivity analysis, subgroup analysis, and univariate meta-regression analysis are employed to deal with high heterogeneity.

A meta-regression analysis is essentially an observational study that uses regression analysis to explore the effect of certain trial or case characteristics (covariates) on the combined effect in a meta-analysis in an attempt to clarify the sources of heterogeneity across studies and to explore the effect of covariates on the combined effect. Univariate meta-regression analyses are conducted to explore the cause of heterogeneity and association between factors including intervention, drug deliver, sample size, mean age, duration and publication year, and the clinical efficacy of mBTWD on radiation enteritis when there are over 10 studies included. Egger's test is a simple quantitative method for testing the symmetry of funnel plots by linear regression, developed by Matthias Egger et al. in 1997 to overcome the shortcomings of the funnel plot method. The theoretical basis of Begg's test is based on Kendall's tau rank correlation method, which determines the existence of “publication bias” by the presence of Kendall's correlation between the standardized effect estimates and the variance of the effect estimates. The trim and fill method aims to identify and correct funnel plot asymmetries caused by publication bias. This method allows both the number of missing studies to be estimated and the inclusion of missing studies to be rerun in a meta-analysis, correcting for the combined effect size of the intervention. Egger's test, Begg's test, and the funnel-plot-based trim and fill method are used to deal with the potential publication bias.

The online Grading of Recommendations Assessment, Development, and Evaluation (GRADE) tool [11] is used to evaluate the quality of evidence (https://gdt.gradepro.org/app/). The GRADE standard is a grading of the body of evidence that takes into account the type of study design, methodological quality, consistency of results, and directness of evidence. It has been adopted by over 100 international organisations and associations worldwide as one of the international standards for evaluating interventional evidence. We mainly use the risk of bias, inconsistency, indirectness, imprecision, and other considerations to evaluate quality. There are four levels of evidence: quality of evidence-high, moderate, low, or very low.

### 2.6. Network Analysis and Molecular Docking

Firstly, the chemical constituents of representative drugs, Baitouweng, Huanglian, Huangbo, and Qinpi, are collected by searching the Traditional Chinese Medicine Systems Pharmacology (TCMSP) database (https://tcmspw.com/tcmsp.php). The main active compounds are obtained with oral bioavailability (OBA) greater than or equal to 30% and drug-like (DL) greater than or equal to 0.18 as screening conditions [12]. Then we screen the target protein corresponding to the active ingredients and convert the target protein name into the corresponding Gene Symbol. The UniProtKB database (https://www.uniporot.org/) is used to check the target information.

Secondly, we set the keywords to “radiation enteritis” to search for the genes related to radiation enteritis from the five sources: GeneCards (https://www.pharmgkb.org/), OMIM (https://omim.org/) and TTD (https://db.idrblab.net/ttd/). All targets associated with radiation enteritis are obtained after repeated genes. The R language program and Venny 2.1 software are used to predict the drug-disease common targets. Then we use the Perl program and Cytoscape 3.8.2 software to build a compound-target-disease network.

Thirdly, common drug-disease targets are imported into the STRING database (https://string-db.org/cgi/input.pl), setting the species as “Homo Sapiens” and the Protein-protein interaction (PPI) network is formed. We then import the PPI network into Cytoscape 3.8.2 software and simplify it according to four topology attributes (DC, Degree Centrality; BC, Between Ness Centrality; CC, Closeness Centrality; EC, Eigenvec Tor Centrality). Thus, we obtain the final core target PPI network.

Next, ClueGO plug-ins are used to conduct Gene Ontology (GO) functional enrichment analysis and Kyoto Encyclopedia of Genes and Genomes (KEGG) pathway analysis for the core targets to screen out the main gene items and signal pathways related to radiation enteritis. GO is divided into three parts: BP (biological process), CC (cellular component), and MF (Molecular Function) [13].

Finally, ligand files and receptor files for molecular docking need to be prepared. (1) Ligand files: 2D structures of small molecule ligands of each core target are downloaded from the PubChem database (https://pubchem.ncbi.nlm.nih.gov/) and are converted from 2D to 3D structures with ChemOffice software. We then optimize them with the AutoDockTools (Version 1.5.6) and save them in PDBQT format as ligand files. (2) Receptor files: the PDB files of the 3D structures of the core target are downloaded from Protein Data Bank (PDB) database (https://www.rcsb.org/). Then we process them with Pymol software (Version 3.2.2) and AutoDockTools software (Version 1.5.6) and save them in PDBQT format as receptor files. (3) Molecular docking: AutoGrid software is used to determine the active pocket of the docking receptor, AutoDock Vina software (Version 1.1.2) is selected for molecular docking, and Pymol software (Version 3.2.2) software is used for analysis and mapping. At the same time, dexamethasone, the commonly used drug in radiation enteritis treatment, is selected as a positive control for analysis and verification.

## 3. Results

### 3.1. Retrieval Results and Study Characteristics

**Figure 2:**
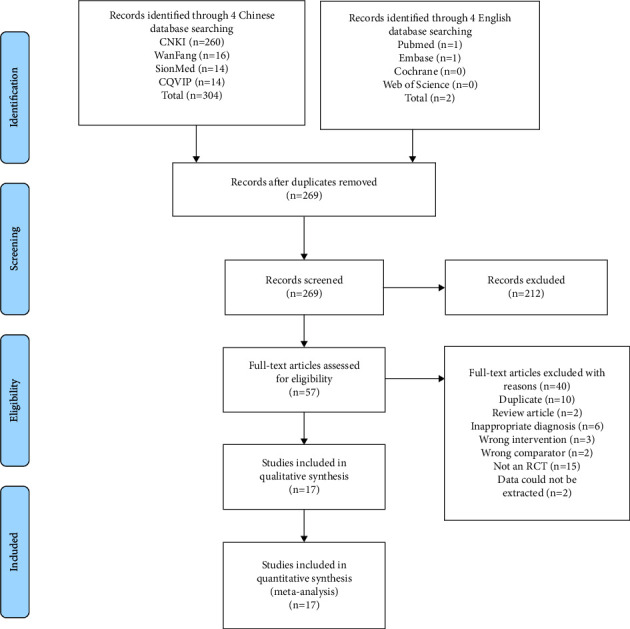
Flow diagram of eligible study selection.

A total of 306 related studies were preliminarily retrieved, 37 duplicate articles were removed, and 212 articles were excluded after reading titles and abstracts. The remaining 57 papers were read in full and 17 studies selected that met the inclusion criteria (Figure 2).

The baseline of the 17 included studies is consistent. The intervention groups receive mBTWD alone or in combination with conventional western medicine. The control groups are given a placebo, gentamicin, dexamethasone, montmorillonite powder, procaine/lidocaine, or combination therapy. The main route of administration is retention enema [9, 14–22]; three studies report the route of administration of retention enema combined with per os [23–25], and four studies report oral administration [26–29]. A total of 1611 patients are enrolled. The baseline characteristics of included trials are shown in Table 1. The compositions of mBTWD from the included studies are shown in Table 2.

### 3.2. Risk of Bias and Quality Assessment

**Figure 3:**
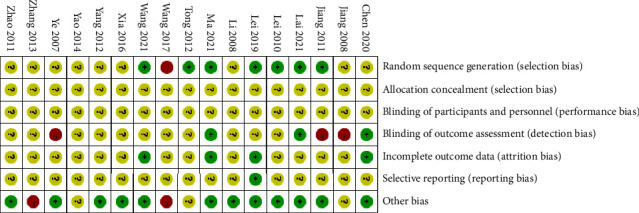
Risk of bias summary.

All of the included studies are randomized controlled trials (RCTs). The risk of bias is assessed according to Cochrane Manual standards. (1) Randomization is mentioned in all included studies, and seven studies [9, 15–17, 25, 27, 29] mention the “random number table.” However, none of the studies mention distribution hiding methods. (2) None of the studies is designed to mention “blindness.” Although blindness is inadequate in the three studies [9, 14, 15], the outcomes are judged by the system evaluators to be mainly objectively detectable indicators and unlikely to be affected by the lack of blindness. (3) Only two studies [9, 16] describe the missing data, and two studies [14, 29] identify no cases of deletions. No absence or exclusion is reported in the remaining studies. (4) Only one study [16] publishes all prestated results, with no selective reporting. The remaining studies could not be evaluated for too few indicators or the absence of project proposals, raising suspicions about selective reporting. (5) Two studies [21, 22] are considered high-risk because of potential sources of bias associated with a particular trial design. As a result, the quality of the included studies is low (Figure 3).

### 3.3. Primary Outcome Measure

#### 3.3.1. Clinical Efficacy

**Figure 4:**
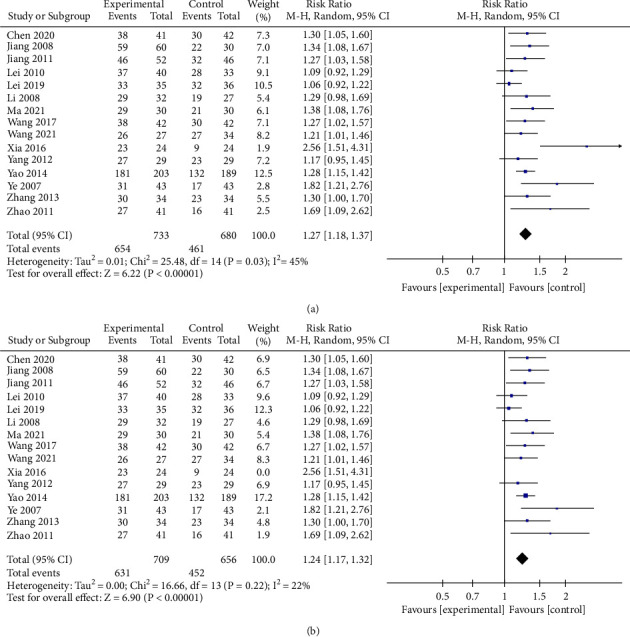
(a and b) Forest plots of the clinical efficacy.

**Figure 5:**
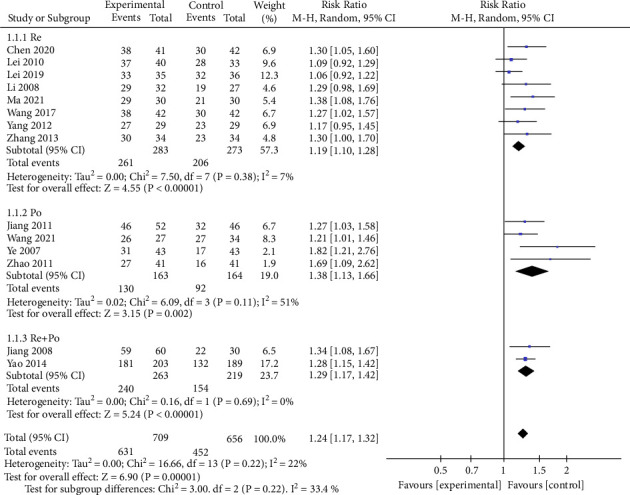
Subgroup analysis of clinical efficacy. Re, retention enema; Po, per os.

Fifteen studies [9, 14, 16–24, 26–29] compare the clinical efficacy of mBTWD with CWM in 1413 patients with radiation enteritis. Due to the moderate heterogeneity of this meta-analysis (Chi^2^ = 25.48, *P*=0.03, *I*^2^ = 45%), a random-effects model is carried out for the meta-analysis to estimate the RR (Figure 4(a)). From the forest plots, we find that the study [18] deviates significantly from the invalid vertical line; after eliminating this study, the heterogeneity decreases (Chi^2^ = 16.66, *P*=0.22, *I*^2^ = 22%) (Figure 4(b)). In addition, we also analyze that differences in drug deliver may be a source of heterogeneity, so we perform a subgroup analysis comprising three subgroups: reserved enema (Re), per os (Po), and Re + Po. The heterogeneity of each subgroup is further reduced (Chi^2^ = 7.5, *P*=0.38, *I*^2^ = 7%; Chi^2^ = 6.09, *P*=0.11, *I*^2^ = 51%; Chi^2^ = 0.16, *P*=0.69, *I*^2^ = 0%) and the differences in each subgroup are statistically significant (*P* < 0.00001; *P*=0.002; *P* < 0.00001). The clinical efficacy of the mBTWD intervention groups is significantly higher than that of the control groups (RR = 1.24, 95% CI (1.17, 1.32), *P* < 0.00001) (Figure 5). The results show that different drug deliver produce heterogeneity to some extent.

Next, we read the full text of the excluded study [18] in detail and found that it lacks clear diagnostic criteria, suggesting that this is a low-quality study and should be excluded.

#### 3.3.2. Total Score of TCM Syndrome

**Figure 6:**
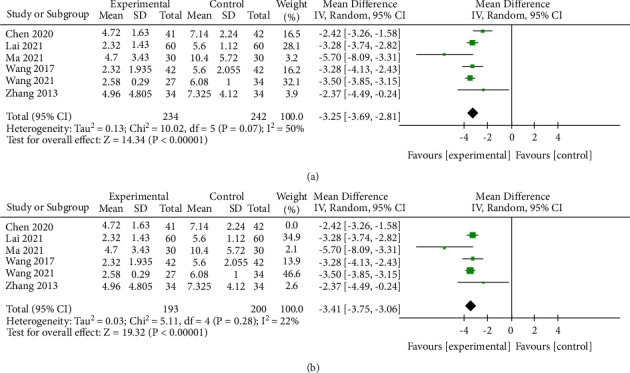
Forest plots of the total score of TCM syndrome. (a) Original data. (b) After eliminating the study with a large heterogeneity.

The main symptom (bellyache, diarrhea, tenesmus, mucosanguineous feces, burning pain in the anus, and so on) of patients is scored according to the quantitative criteria of TCM syndrome (SFDA, 2002): 0 = none, 1 = mild, 2 = moderate, 3 = severity. Moreover, Stata 14.0 software is used to combine effect sizes. A total of 7 studies describe TCM syndrome's total score regarding this standard, one study [16] is excluded from describing the discrete variables. The remaining 6 studies [9, 14, 15, 21, 22, 29] include a total of 476 patients. The results of the meta-analysis show a moderate heterogeneous in the total score of the TCM syndrome (Chi^2^ = 10.02, *P*=0.07, *I*^2^ = 50%) (Figure 6(a)). It can be seen from the forest plots that the study [14] has the least overlap with other studies; then a sensitivity analysis is conducted and heterogeneity is significantly reduced after this study is removed (Chi^2^ = 5.11, *P*=0.28, *I*^2^ = 22%) (Figure 6(b)). After careful reading of this study, we find that the score of systemic symptoms is included, while other studies are mainly based on the local symptom of the intestinal tract. Therefore, we believe that this is due to heterogeneity caused by differences in trial design, so it is excluded.

According to the results of the meta-analysis, the difference in the TCM syndrome total score between the two groups after treatment is statistically significant (MD = −3.41, 95% CI (−3.75, −3.06), *P* < 0.00001) (Figure 6(b)). Therefore, we discover that mBTWD has certain advantages in improving bellyache, diarrhea, tenesmus, mucosanguineous feces, and burning pain in anus syndromes.

#### 3.3.3. Colonoscopy Score/Grade and Radiation Enteritis Grading

**Figure 7:**
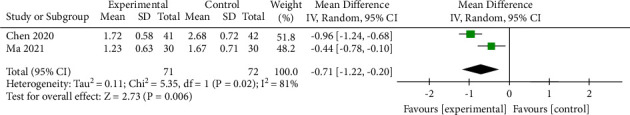
Forest plots of colonoscopy score.

Two studies [9, 14] report the comparison of the colonoscopy score of radiation enteritis patients between the mBTWD and CWM groups. The results show a high heterogeneity (Chi^2^ = 5.35, *P*=0.02, *I*^2^ = 81%) (Figure 7). After careful reading of that two studies, we find the heterogeneity may come from different scoring criteria. Instead of a random-effects model, there is a statistically significant difference between the two groups (MD = −0.71, 95% CI (−1.22, −0.20), *P*=0.006) (Figure 7).

**Figure 8:**
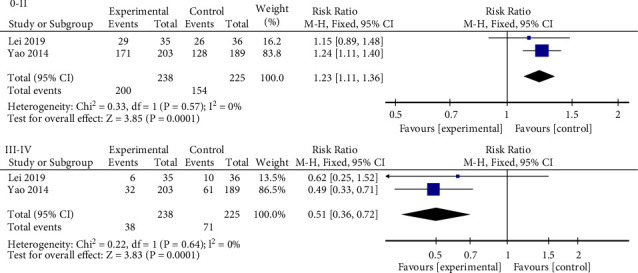
Forest plots of colonoscopy grade (0–IV degree). 0–II, the injury of the intestinal mucosa is significantly improved or restored to normal; III-IV, the injury of the intestinal mucosa is serious or irreversible.

Two studies [16, 24], with a total of 463 patients, compare the efficacy of mBTWD alone with combined CWM by estimating the grade of colonoscopy. According to the Gareau classification criteria of Gareau et al. [30], intestinal mucosal injury under colonoscopy is divided into degrees 0 to IV: 0-II, intestinal mucosal injury is significantly improved or normalized; III to normal; III-IV, the injury of the intestinal mucosa is serious or irreversible. Meta-analysis is conducted according to 0-II and III-IV, respectively, and the results show good homogeneity between studies of different grades (Chi^2^ = 0.33, *P*=0.57, *I*^2^ = 0%; Chi^2^ = 0.22, *P*=0.64, *I*^2^ = 0%) (Figure 8). Moreover, there is a significant difference in colonoscopy grade between the two groups after treatment (RR = 1.23, 95% CI (1.11, 1.36), *P*=0.0001; RR = 0.51, 95% CI (0.36, 0.72), *P*=0.0001) (Figure 8).

**Figure 9:**
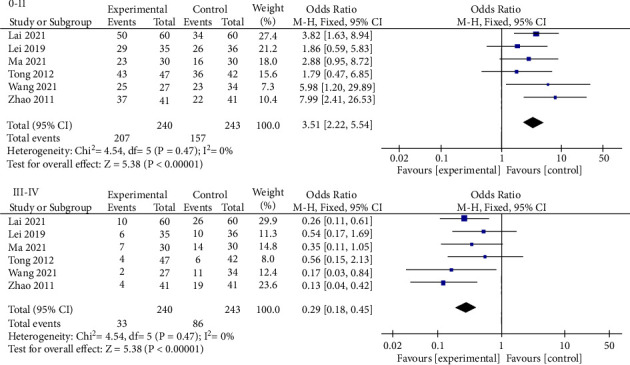
Forest plots of radiation enteritis classification (0, IV degree). 0-II, the intestinal reaction is significantly improved or restored to normal; III-IV, the intestinal reaction is serious or irreversible.

Six studies [9, 15, 16, 25, 26, 29], with a total of 483 patients, report the comparison of radiation enteritis grading between the mBTWD and CWM groups. According to the RTOG/EORCT radiation injury classification scheme [31], the intestinal reaction after radiotherapy is classified into degrees 0–IV: 0–II, the intestinal reaction is significantly improved or restored to normal; III-IV, the intestinal reaction is serious or irreversible. The meta-analysis is conducted according to 0-II and III-IV, respectively; there is little heterogeneity among these studies at different grades (Chi^2^ = 4.54, *P*=0.47, *I*^2^ = 0%; Chi^2^ = 4.54, *P*=0.47, *I*^2^ = 0%) (Figure 9). Moreover, there is a significant difference in radiation enteritis grading between the two groups after treatment (OR = 3.51, 95% CI (2.22, 5.54), *P* < 0.00001; OR = 0.29, 95% CI (0.18, 0.45), *P* < 0.00001) (Figure 9).

Overall results show that mBTWD can significantly improve intestinal mucosal injury and reduce the degree of intestinal reaction after radiotherapy.

#### 3.3.4. C-Reactive Protein Level

**Figure 10:**
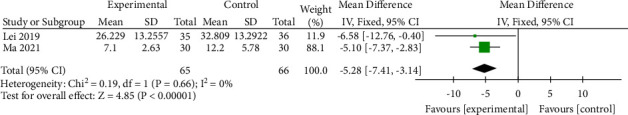
Forest plots of CRP level. CRP, C-reactive protein.

Two studies [9, 16] report a comparison of C-reactive protein (CRP) levels between groups, with both control groups treated with 0.9% NaCl solution 100 ml + dexamethasone 10 mg + montmorillonite powder 6 g. No heterogeneity is found in the results (Chi^2^ = 0.19, *P*=0.66, *I*^2^ = 0%) (Figure 10). Therefore, with a fixed effect model, there is a significant difference in mBTWD versus CWM (MD = −5.28, 95% CI (−7.41, −3.14), *P* < 0.00001) (Figure 10).

#### 3.3.5. Karnofsky Performance Scale

**Figure 11:**
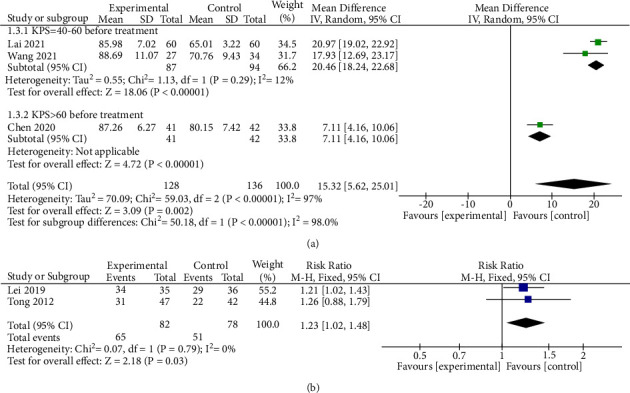
Forest plots of KPS score. KPS, Karnofsky. (a) Continuous variable. (b) Dichotomous variable.

Five studies report the comparison of the Karnofsky performance scale (KPS) score [32] among mBTWD and CWM groups. Three of these studies [14, 15, 29] with a total of 264 radiation enteritis patients, describing continuous variables. As shown in Figure 11(a), there is a high heterogeneity in the results (Chi^2^ = 59.03, *P* < 0.00001, *I*^2^ = 97%). Then, a subgroup analysis is performed due to differences in KPS score before treatment: KPS = 40–60 and KPS > 60 before treatment. The results show that heterogeneity decreased significantly after the subgroup analysis (Chi^2^ = 1.13, *P*=0.29, *I*^2^ = 12%). Moreover, the KPS score is significantly different between the two groups (MD = 15.32, 95% CI (5.62, 25.01), *P*=0.002) (Figure 11(a)).

Two studies [16, 25] with a total of 160 patients report the number of patients with a KPS greater than 80 after treatment, which is a dichotomous variable. There is little heterogeneity in the results (Chi^2^ = 0.07, *P*=0.79, *I*^2^ = 0%) (Figure 11(b)). And the difference between the two groups is statistically significant (RR = 1.23, 95% CI (1.02, 1.48), *P*=0.03) (Figure 11(b)).

#### 3.3.6. Fecal Occult Blood

**Figure 12:**
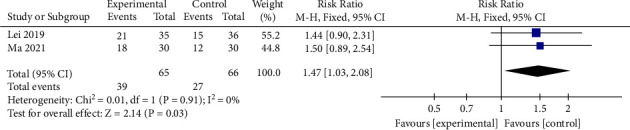
Forest plots of fecal occult blood.

Two studies [9, 16] with a total of 131 patients report the comparison of occult fecal blood (OB) between mBTWD and the CWM groups. We mainly analyze the number of patients with negative or weakly positive fecal occult blood tests (OB: 0∼+) after treatment. There is slight heterogeneity in the results (Chi^2^ = 0.01, *P*=0.91, *I*^2^ = 0%) (Figure 12). Hence, the improvement of fecal occult blood is significantly different between the two groups (RR = 1.47, 95% CI (1.03, 2.08), *P*=0.03) (Figure 12).

#### 3.3.7. Safety

No serious adverse events occur in nearly all of the studies, and there is no significant difference in the incidence of adverse events between the intervention groups and the control group.

**Figure 13:**
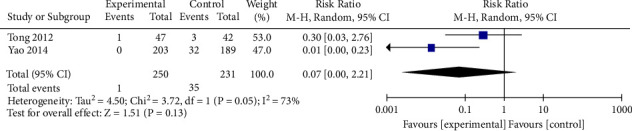
Forest plots of recurrence rate.

Two studies with 481 radiation enteritis patients that compared mBTWD with CWM are identified in this analysis [24, 25]. The heterogeneity between the two studies is large (Chi^2^ = 3.72, *P*=0.05, *I*^2^ = 73%) (Figure 13). Hence, the difference in the rate of recurrence between the two groups is not statistically significant (RR = 0.07, 95% CI (0.00, 2.21), *P*=0.13) (Figure 13).

### 3.4. Univariate Meta Regression

**Figure 14:**
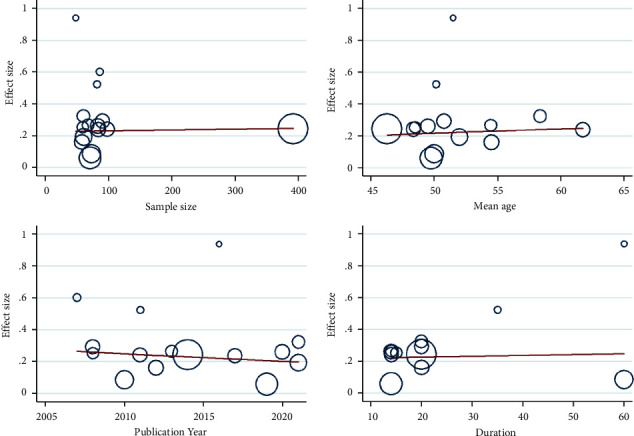
Meta-regression of clinical efficacy with continuous covariates.

Since more than 10 studies are included in the analysis of clinical efficacy, we conduct a univariate meta regression here to explore the association between the clinical efficacy indicator and the difference in intervention, drug delivery, sample size, publication year, or other characteristics of the studies including the mean age and duration of treatment. Both dichotomous and continuous covariates are employed in the regression models; the results of the univariate meta-regression analyses are presented in Table 3 and Figure 14. It is found that the association with the effect size of the intervention (mBTWD with CWM) on the clinical efficacy of mBTWD in the treatment of radiation enteritis is not statistically significant (meta-regression coefficient 1.014, CI 0.859, 1.198, *P*=0.852, *I*^2^ = 39.730%, Tau^2^ = 0.328%), suggesting that the difference in the intervention is unlikely to increase clinical efficacy with the baseline levels. Then none of the coefficients of other covariates are significantly associated with the estimated risk ratio, and for this specific reason, multivariate analyses are not performed.

### 3.5. Publication Bias

**Figure 15:**
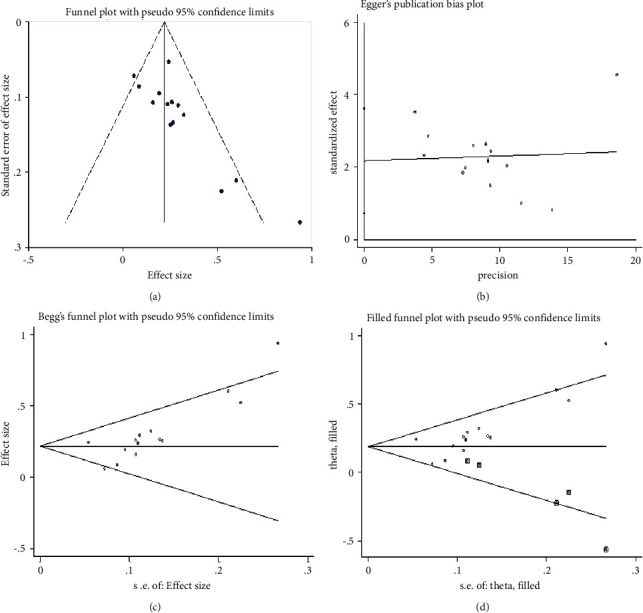
Funnel plots of clinical efficacy of mBTWD in treating radiation enteritis. (a) Basic funnel plot. (b) Egger's funnel plot. (c) Begg's funnel plot. (d) Filled funnel plot of metatrim analysis.

Since most clinical outcome variables contain less than 10 studies, performing the funnel plot could be of little significance, so it is difficult to fully identify the risk of publication bias. Specifically, the meta-analysis of the clinical efficacy indicator contained up to 15 eligible studies for us to conduct a Funnel plot, as shown in Figure 15(a), visual inspection of the funnel plot suggests a little asymmetry, indicating the existence of publication bias.

Furthermore, we accurately perform publication bias using Egger's test (Figure 15(b)) and Begg's test (Figure 15(c)). All analyses are performed with Stata 14.0 software and results are shown with 95% confidence intervals. Both Egger's test and Begg's test detect the existence of publication bias (coefficient = 2.175, *P*=0.006), indicating that the validity and generalization of our conclusions would be limited and affected due to possible publication bias.

Thus, we try to employ another Funnel-plot-based trim and fill method to deal with the potential impact of publication bias, as shown in Figure 15(d). In the trim and fill method, we can re-estimate the actual effect size by filling in the “missing” studies and forming a new pooled estimate until the funnel plot reaches a new symmetry. After 4 iterations, the procedure identifies and trims 4 studies (4 inserted studies as their theoretical counterparts) until the distribution is symmetrical, with the overall effect size estimated as RR = 1.21 (95% CI from 1.12 to 1.31, *P* < 0.001). Compared with our initial pooled effect size of RR = 1.27, which is substantially larger than the bias-corrected effect size and indicates that the potential publication bias made the initial results overestimated (approximately 4.9%), the real effect when controlling for selective publication bias could be slightly lower. This indicates that our results are still robust even with the occurrence of publication bias.

### 3.6. Assessment Quality of Evidence

Several types of evidence for mBTWD in the treatment of radiation enteritis are included in our meta-analysis; the GRADE evidence rating levels performed with the online tool are shown in Table 4. The evidence quality for clinical efficacy and recurrence rate is rated as very low due to serious clinical or statistical heterogeneity problems in risk of bias, inconsistency, imprecision, or publication bias. The quality of the evidence for KPS is rated low with other conditions rated moderate. The criteria and reasons for upgrading or lowering the quality of evidence for each outcome are as follows. (1) Study design: all studies included in this paper are randomized controlled trials that satisfied our inclusion criteria. (2) Risk of bias: although sensitivity analysis that excludes trials with a high risk of bias does not change the main results, all of these studies are downgraded due to a lack of blinding. (3) Inconsistency: high statistical heterogeneity (*I*^2^ > 50%) occurrence will downgrade the evidence quality to a lower level; three outcomes are marked as serious inconsistency. (4) Indirectness: mBTWD is implemented in the treatment of radiation enteritis and is directly related to those clinical outcomes in our study, so there is no downgrade of evidence. (5) Imprecision: the evidence will be downgraded if the 95% CI crosses no treatment effect or if the estimated effect size is significantly different (*P* > 0.05). (6) Other Considerations: downgrades if serious publication bias is detected to be significant (*P* < 0.05%), which occurs in the clinical efficacy analysis.

### 3.7. Sensitivity Analysis

**Figure 16:**
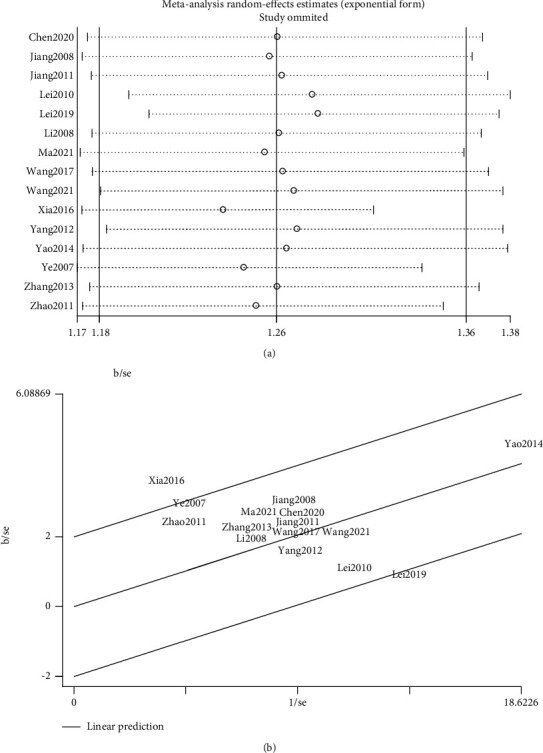
Sensitivity analyses of the clinical efficacy of mBTWD in treating radiation enteritis. (a) The one-by-one dropped out estimation results. (b) The Galbraith plot.

With evidence of the publication bias, we also propose sensitivity analyses to investigate the potential causes of heterogeneity and identify unbalanced or disproportionate contributions to the observed bias from these trials. Sensitivity analyzes are performed using *metainf* command with Stata 14.0, by repeating the baseline meta-analysis by excluding assumed “biased” trials one-by-one to assess their impact on the overall estimate; relevant results are shown in Figure 16(a). It is shown that dropping out trials one by one may have little impact on the overall estimated effect size while expanding the confidential levels from (1.18, 1.36) to (1.17, 1.38). Among those excluded studies, dropping Xia [18] and Ye and Wang [28] may lead to a lower effect size while excluding Lei [17] and Lei [16] could cause a higher effect size. For better visual inspection, we also introduce the Galbraith plot to identify possible outlier studies that have an excessive influence on the overall estimate (Figure 16(b)). From the Galbraith plot, we could draw a similar conclusion that these four studies [16–18, 28] are the main possible outliers with a higher risk of heterogeneity and can be correlated with publication bias.

### 3.8. Network Analysis and Molecular Docking Results

#### 3.8.1. Active Ingredients and Targets Screening Results

**Figure 17:**
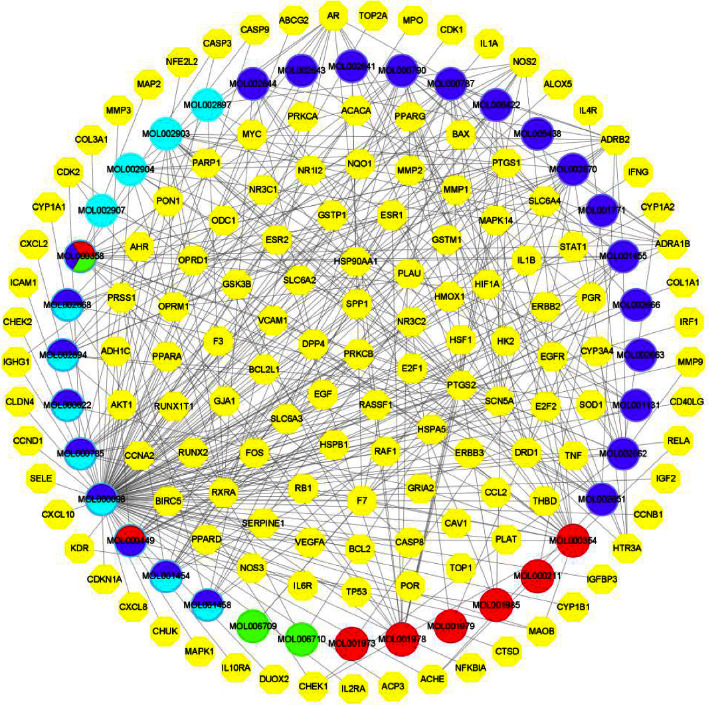
Network of active ingredients-targets. Yellow: human-derived target proteins. Red: Baitouweng. Purple: huangbo. Light blue: huanglian. Green: Qinpi. Mixed color: multidrug.

According to the OB and DL characteristics of the compounds, a total of 65 active ingredients are obtained from the TCMSP database; there are 11 in Baitouweng (BTW), 37 in Huangbo (HB), 14 in Huanglian (HL), and 3 in Qinpi (QP). After removing the duplicate ingredients, a total of 51 are left (Table 5). Then, we screen the corresponding targets of the active ingredients, and 987 human-derived target proteins are obtained after the duplicate targets are removed. The Cytoscape 3.8.2 software is used to connect the active ingredients-targets network. The network consists of 175 nodes and 381 edges, these nodes represent compounds and the corresponding targets, and edges represent interactions between compounds and target proteins (Figure 17).

#### 3.8.2. Prediction and Construction of Drug-Disease Networks

**Figure 18:**
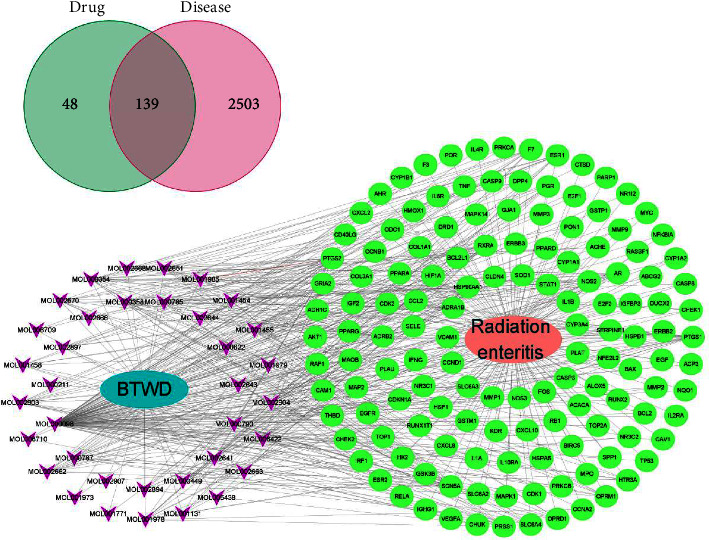
Network of BTWD active ingredients-targets-radiation enteritis.

Five databases (OMIM, GeneCards, PharmGkb, DrugBank, and TTD) are searched to obtain 2642 disease targets related to radiation enteritis. Using the R language program and Venny2.1 software, the intersection of mBTWD and radiation enteritis targets is selected, and finally 139 common drug-disease targets are screened out. Finally, Cytoscape 3.8.2 software is used to build the mBTWD active ingredients-targets-radiation enteritis network (Figure 18). Of these, quercetin (MOL000098) interacted with 114 targets, isorhamnetin (MOL000354) interacted with 21 targets, and beta-sitosterol (MOL000358) interacted with 18 targets. Other compounds, such as stigmasterol (MOL000449) and aureusidin (MOL001978), are associated with multiple targets, suggesting that the compounds in BTWD may exert pharmacological effects against radiation enteritis by acting together on these targets.

#### 3.8.3. PPI Network of Key Targets

**Figure 19:**
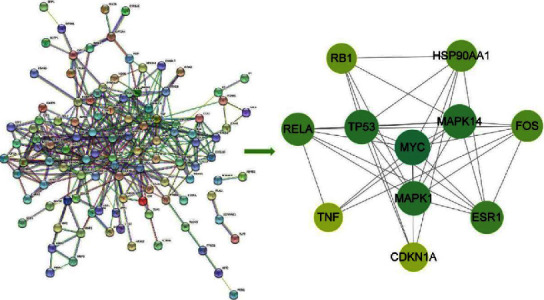
PPI network of the key targets for representative drugs in mBTWD against radiation enteritis.

These 139 drug-disease common targets are imported into the STRING database, and the PPI network of targets for mBTWD against radiation enteritis could be obtained by removing the noncorrelated targets. Then the PPI network is imported into Cytoscape 3.8.2 software, and the CytoNCA plug-in is used to calculate the median value of network nodes. Finally, eleven key targets are obtained, and the PPI network of core targets is constructed. The larger the node, the darker the color, and the higher the DC value (Figure 19). From the network, we can see that the top four are MYC, TP53, MAPK14, and MAPK1, and their DC values are 9, 8, 8, and 8, respectively. These results indicate that these targets are the key targets of BTWD in the treatment of radiation enteritis.

#### 3.8.4. GO and KEGG Enrichment Analysis of Key Targets

**Figure 20:**
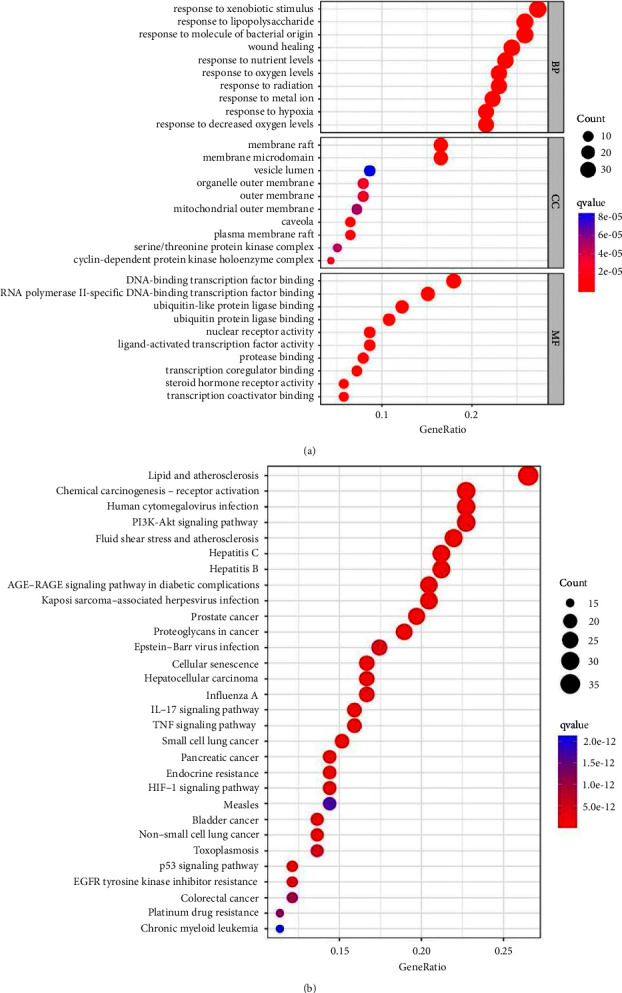
GO and KEGG enrichment analysis of the key targets for mBTWD against radiation enteritis. (a) GO functional annotation. (b) KEGG signaling pathways.

The ClueGO plug-in is used to perform GO functional annotation and KEGG signal pathways enrichment analysis for the key targets of BTWD in the treatment of radiation enteritis (filter criteria *P* < 0.05). The GO enrichment analysis mainly refers to biological process (BP), and a total of 2183 GO items are obtained, mainly involving response to lipopolysaccharide, response to xenobiotic stimulus, and response to molecule of bacterial origin and wound healing (Figure 20(a)). A total of 172 signal pathways are identified in the KEGG enrichment analysis, which are found in the lipid and atherosclerosis, PI3K-Akt signaling pathway, and chemical carcinogenesis-receptor activation (Figure 20(b)).

**Figure 21:**
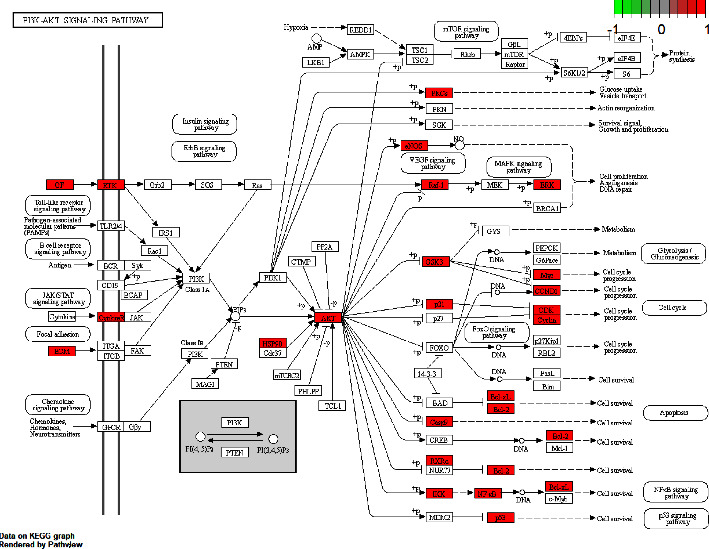
PI3K-Akt signaling pathway.

According to the results of the KEGG enrichment analysis, we select the “hsa0415” (PI3K-Akt signaling pathway) to draw a pathway map using the Pathview plug-in. And the red nodes indicate that the key target genes exist in the regulatory network (Figure 21).

#### 3.8.5. Molecular Docking of Active Ingredients and Key Targets

**Figure 22:**
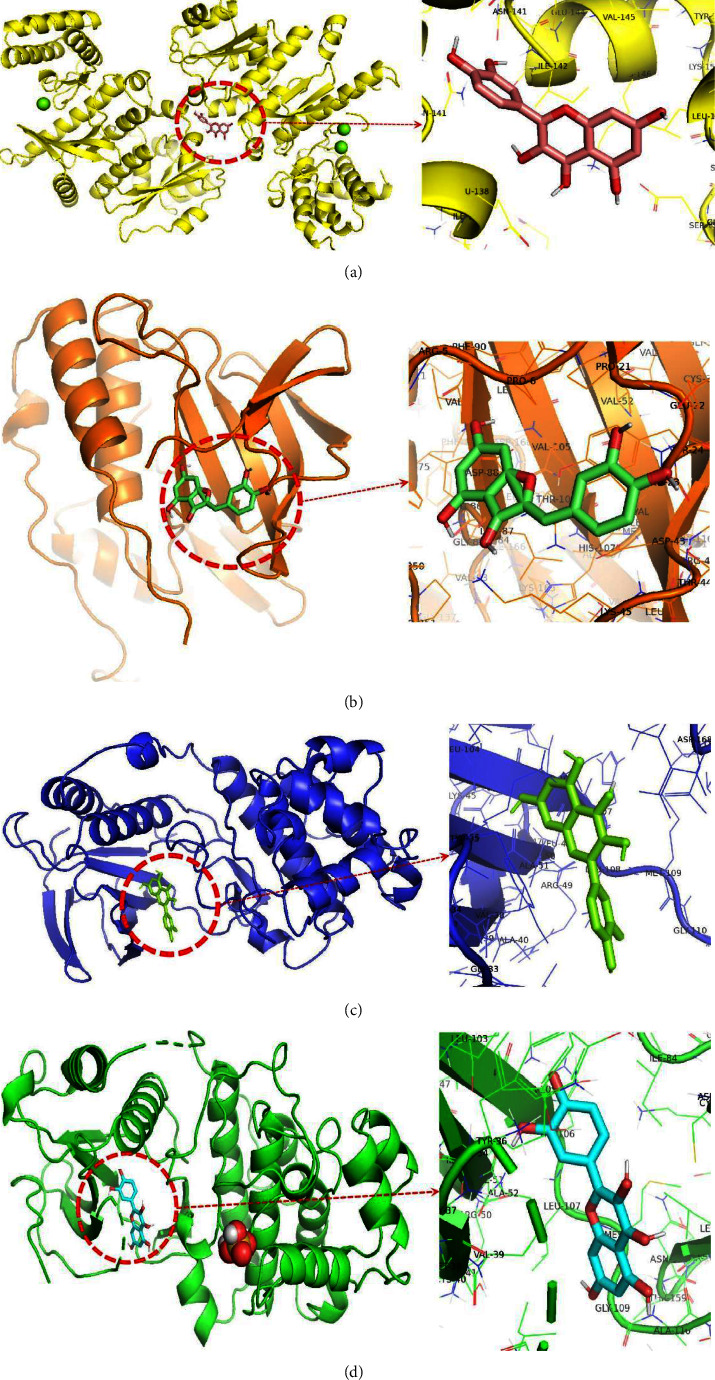
Three-dimensional structure diagram of molecular docking. (a) TP53-quercetin. (b) MAPK14- aureusidin. (c) MAPK14-isorhamnetin. (d) MAPK1-quercetin.

According to the PPI network, the key targets of mBTWD in the treatment of radiation enteritis are MYC, TP53, MAPK14, and MAPK1. Molecular coupling is performed for the 4 core targets with the active ingredients of mBTWD and the positive control drug dexamethasone. Affinity < −5.0 kJ·mol^−1^ indicated good binding activity between ligands and receptors [33]. The results show that MAPK1 has the highest binding activity with quercetin (affinity = −8.5 kJ·mol^−1^), followed by TP53 with quercetin (affinity = −8.3 kJ·mol^−1^). MAPK14 with aureusidin (affinity = −7.7 kJ·mol^−1^), MAPK14 with isorhamnetin (affinity = −7.2 kJ·mol^−1^). Additionally, the binding activity of these active ingredients with the key targets is stronger than the positive control drug dexamethasone (Table 6). The optimal molecular docking diagram is shown in Figure 22.

## 4. Discussion

### 4.1. Summary of the Evidence and Results

Radiation enteritis is a common side effect of radiotherapy in patients with pelvic/abdominal malignancy. The small intestine is quite sensitive to radiation, and radiotherapy could easily cause intestinal wall damage, leading to inflammatory infiltration of intestinal epithelial cells. Patients present with an intolerable change in stool habits that last for a long time, causing the discontinuation of radiotherapy, which severely impair their quality of life and reduce survival [34]. Acute radiotoxicity mainly damages the intestinal mucosa, leading to a decrease in the normal intestinal villous epithelial barrier and presenting as abdominal pain. Chronic radiation toxicity primarily affects the muscular and serosal layers, causing vascular degeneration and fibrosis, and manifests itself as chronic diarrhea and malabsorption, the formation of ischemic intestinal disease, intestinal flora disorder, and chronic enteritis, followed by an intestinal mucosal ulcer, perforation or abscess, and finally, the formation of intestinal obstruction and microbial over-propagation [35, 36]. Evidence also suggests that gut microbiota dysbiosis plays an important role in the development of radiation enteritis, and it is a reminder that radiation injury can be relieved by modifying the local microecosystem [1, 2]. Therefore, some formulae of Chinese medicine with an anti-inflammatory effect, such as Baitouweng decoction, may be a complementary means for recurrent radiation enteritis that is difficult to control by conventional western medicine.

The results of this meta-analysis show that mBTWD alone or in combination with CWM can benefit patients with radiation enteritis with better clinical efficacy than CWM alone. Due to the moderate heterogeneity (*P*=0.03, *I*^2^ = 45%), we eliminate the study [18] for its unclear diagnostic criteria by sensitivity analysis. In addition, subgroup analysis is performed according to different drug deliver (Re, Po, and Re + Po) to identify possible sources of heterogeneity; we find that the heterogeneity of these subgroups are decreased (*P*=0.38, *I*^2^ = 7%; *P*=0.11, *I*^2^ = 51%; *P*=0.69, *I*^2^ = 0%), this indicates that there is indeed some heterogeneity in different drug deliver. In light of the *P* value results of Re and Po (*P* < 0.00001; *P*=0.002), we believe that the difference in efficacy of retention enema seems to be more significant. This might be due to the reason the TCM decoction can be quickly absorbed into the blood through the intestinal mucosa after the enema, acting directly on the lesions and avoiding the elimination of drugs by the hepatoenteric circulation [37]. To sum up, based on the remaining 14 studies, we have reason to think that mBTWD has a good clinical effect on radiation enteritis, and the effect can be increased by about 24% compared with the control groups (RR = 1.24, *P* < 0.00001). A study comparing montmorillonite powder alone or combined with dexamethasone in the treatment of acute radiation enteritis reports that the clinical efficacy of the two groups is 72.09% and 97.67%, respectively [38]. While the results of the meta-analysis in this article show that the mean clinical efficacy of mBTWD combination therapy is approximately 89.2%. This suggests that for the treatment of acute radiation enteritis, mBTWD combined with dexamethasone can be effective in relieving symptoms.

mBTWD combination therapy can significantly improve the symptoms of TCM. Compared to western medicine treatment alone, mBTWD combination therapy alleviates symptoms of bellyache, diarrhea, tenesmus, and mucosanguineous feces in patients with radiation enteritis, and the total score of the TCM syndrome decreases by an average of 3.41 points (MD = −3.41, *P* < 0.00001). Furthermore, mBTWD could improve the local bowel symptoms in patients. The intestinal mucosal improvement rate in the mBTWD groups is about 23% higher than that in the CWM groups (RR = 1.23, *P*=0.0001), correspondingly, the deterioration rate decreases by nearly half compared to the control groups (RR = 0.51, *P*=0.0001). Colonoscopy objective signs are reported in four studies, two of which report colonoscopic intestinal mucosa scoring and another two are grading. However, these two studies of continuous variables show great heterogeneity due to different scoring criteria (*P*=0.02, *I*^2^ = 81%), and sensitivity analysis are unable to be conducted due to the small sample size. Another two studies estimate the grade of colonoscopy (0–IV degree) according to the same criteria, both 0-II and III-IV have strong homogeneity (*P*=0.57, *I*^2^ = 0%; *P*=0.64, *I*^2^ = 0%). Another 6 studies also report the subjective symptoms of radiation enteritis grading (0–IV degree), in order to reduce the heterogeneity, we select the OR for statistical analysis, both 0–II and III-IV have strong homogeneity (*P*=0.47, *I*^2^ = 0%; *P*=0.47, *I*^2^ = 0%). The improvement rate of the radiation enteritis classification in the mBTWD groups is 3.51 times higher than that in the CWM groups (OR = 3.51, *P* < 0.00001); correspondingly, the deterioration rate is only 0.29 times that of the CWM groups (OR = 0.29, *P* < 0.00001). Similarly, we compare the findings obtained with previous studies and the data show a higher efficacy of colonoscopy after mBTWD treatment compared to montmorillonite alone (82.03 vs. 65.12%) [38]. These results indicate that, in general, mBTWD has a certain positive effect on the improvement of intestinal mucosal signs and symptoms and is significantly correlated with the reduction of the rate of deterioration.

Only two studies with a total of 161 samples report the CRP level, there is no heterogeneity in the results (*P*=0.66, *I*^2^ = 0%), and the CRP level decreases by an average of 5.28 mg/L compared with the control groups after treatment (MD = −5.28, *P* < 0.00001). Five studies report the KPS score, including three continuous variables and two dichotomies: for the continuous variables, a subgroup analysis is performed according to the KPS score before treatment (KPS = 40–60 and KPS > 60) and the results are fine (*P*=0.29, *I*^2^ = 12%), after treatment, the KPS score improved by an average of 15.32 points compared to the control groups (MD = 15.32, *P*=0.002); for dichotomies variables, there is also little heterogeneity (*P*=0.79, *I*^2^ = 0%) and the rate of improvement of life quality in the mBTWD groups is about 23% higher than that of the CWM groups (RR = 1.23, *P*=0.03); these results indicate that mBTWD is effective in improving the quality of life of patients with radiation enteritis. Although only 2 studies report the fecal occult blood, the improvement rate is about 47% higher than the control groups (RR = 1.47, *P*=0.03), suggesting that mBTWD have better hemostasis. Due to fewer included studies and small sample size, we think that the credibility of these meta-analysis results of CRP levels, KPS score, and fecal occult blood is not high.

We are also interested in safety except for efficacy. Fortunately, no serious adverse events occur in both the intervention and control groups, indicating that both mBTWD and conventional western medicine treatments are safe and reliable. However, some studies have reported a certain recurrence rate after treatment, but the difference is not statistically significant between the two groups (RR = 0.07, *P*=0.13). As noted previously, due to the small sample size and the low quality of the evidence, adverse events and recurrence rates also need to be further confirmed. In the future, larger sample sizes and more studies are needed to reach true scientific conclusions.

Based on Egger's test, Begg's test, and Funnel-plot-based trim and fill method, we find that there is indeed publication bias in the reports of clinical efficacy in existing studies; the potential publication bias makes the initial results overestimated by about 4.9%. This indicates that our results are still robust even with the occurrence of publication bias. According to the results of the GRADE evidence, the included overall quality of the studies is low, mainly for the reason that almost all the studies do not follow the double-blind rule and there is publication bias. With the evidence of publication bias, we also propose sensitivity analyses to investigate the potential causes of heterogeneity. The results show that the heterogeneity decreased significantly after eliminating some individual studies one by one, the evidence quality of these studies is relatively low and most of them lacked rigorous and clear inclusion or exclusion criteria.

### 4.2. Potential Molecular Mechanism

The results of network analysis show that BTWD could act on radiation enteritis with multiple targets, components, and pathways. A total of 51 ingredients are discovered and 139 common targets are screened out, such as quercetin, isorhamnetin, and beta-sitosterol, which could reduce the level of inflammatory factors, a powerful antioxidant, endothelial cell protection, and antitumor effects [39–42]. These components may be the key factors in the treatment of radiation enteritis. From the PPI network, the top four are MYC, TP53, MAPK14, and MAPK1; both TP53 and MAPK regulated many biological processes, including apoptosis, protein biosynthesis, oncogenesis, and the cell cycle [43, 44], indicating that these targets are the key targets of BTWD in the treatment of radiation enteritis. GO and KEGG enrichment analysis results also mainly involve some microbial infection and antitumor biological process which are related to the incidence of radiation enteritis. Molecular docking technology further verified the better binding activity of main active ingredients and key targets in BTWD from a quantitative perspective, even better than that of positive control drugs. However, these conclusions are only the results of network pharmacological prediction and lack of further molecular validation.

### 4.3. Limitations and Prospects for Future Research

This study also has several limitations. (1) The research has design flaws, the quality of evidence in existing studies is generally low without involving a specific randomized control grouping method, allocation concealment, blinding, and there is certain publication bias. (2) The included RCT studies are conducted only in China with small sample sizes and lacking larger sample studies in other languages, resulting in low-quality evidence. (3) The inclusion criteria and outcomes evaluation of some studies are not unified, and the course of radiation enteritis is not clearly defined, leading to an increased risk of heterogeneity. (4) There are too few reports on adverse events and recurrence rate, which could not objectively present the safety problems of mBTWD, and there may be false negative results. (5) The composition of mBTWD varies greatly and is not rigorous enough, the addition or subtraction of mBTWD contains many other herbs, and the dosage of mBTWD has not been unified or standardized, which made it difficult to determine the relationship between its efficacy and other ingredients or doses. (6) Network analysis and molecular docking techniques can only predict the possible targets and signaling pathways of representative drugs in mBTWD treatment of radiation enteritis, lacking further molecular verification, and the molecular mechanism needs further demonstration.

With the limitations of current studies, future studies are expected to strictly follow the randomly assigned, blinded, allocation concealment principles or to improve the quality of studies by increasing sample sizes, uniforming inclusion criteria, or outcomes measuring standards. In future studies, it is necessary to define the main components of mBTWD clearly and restrict the dose range to reduce heterogeneity. Based on the results of this meta-analysis, we recommend herbs like Baitouweng, Huanglian, Huangbo, Qinpi, and Diyu be chosen for the clinical application of mBTWD in the treatment of radiation enteritis, and the dosage of each drug could also be adjusted accordingly. Moreover, more detailed information, including withdrawal, adverse events, recurrence rate, and follow-up times, should be recorded and discussed adequately. In particular, the results based on the network analysis and molecular docking indicate that the most probable active ingredients or targets should be experimentally validated to clarify the potential pharmacological mechanisms.

## 5. Conclusion

Although this meta-analysis provides relatively poor quality evidence to validate the efficacy and safety of mBTWD in the treatment of radiation enteritis, the clinical significance of this study lies in two aspects. It provides both an option and an idea for radiation enteritis treatment. In addition, it further supports the unique advantages and usage of traditional Chinese medicine to relieve symptoms and improve the quality of life in cancer patients. However, there are still some limitations. The risk of adverse events and recurrence rate is under-reported, and further experiments should be performed to validate the predicted ingredients and targets. Therefore, it is necessary to design scientifically rigorous large-sample RCTs and supplement basic studies to evaluate the clinical evidence and molecular mechanism of mBTWD in the future treatment of radiation enteritis.

## Figures and Tables

**Table 1:** Basic characteristics of included studies.

First-author(year)	Sample size	Age (year)	Tumor types	Randomised methods	Diagnosis standard	Drug deliver	Intervention	Control	Duration (day)	Outcomes
Chen 2020 [14]	41/42	49.21 ± 8.25/49.74 ± 9.71	Cervical cancer	NR	CCDTRP (2018)	reserved enema	mBTWD (50 ml, qd) + A + rhGM-CSF	A + rhGM-CSF (50 ml, qd)	14	(1), (2), (3), (4), (5)
Zhao 2011 [26]	35/36	49.5 ± 7.6/50.8 ± 7.9	Pelvic tumor	NR	NOHS (2002)	Po	mBTWD (150 ml, bid)	Blank control	35	(1), (3)
Jiang 2008 [23]	60/30	24–78/25–76	Gynecological cancer	NR	SDTMTC	Re + Po	mBTWD (50 ml, qd, Re) + (100 ml, bid, Po)	B (50 ml, qd)	20	(1)
Lai 2021 [15]	60/60	48.6 ± 7.3/50.3 ± 8.6	Cervical cancer	NRT	CCDTRP (2018)	Re	mBTWD (100 ml, bid)	B (100 ml, bid)	20	(2), (3), (4), (7)
Lei 2019 [16]	35/36	49.80 ± 11.66/49.69 ± 11.22	Pelvic tumor	NRT	NOHS (2002)	Re	mBTWD (100 ml, qd)	C (100 ml, qd)	14	(1), (2), (3), (4), (5), (6), (7)
Lei 2010 [17]	40/33	40–60	Cervical cancer	NRT	MOR (2001)	Re	mBTWD (150 ml, qd)	A (150 ml, qd)	60	(1)
Xia 2016 [18]	24/24	37–68/36–65	Cervical cancer	NR	NR	Re	mBTWD (100 ml, qd)	B (100 ml, qd)	60	(1)
Yang 2012 [19]	29/29	34–73/36–75	Pelvic tumor	NR	NR	Re	mBTWD (100 ml, qd)	B (100 ml, qd)	20	(1)
Jiang 2011 [27]	52/46	48.9 ± 7.8/47.8 ± 8.5	Cervical cancer	NRT	MOR (2001)	Po	mBTWD (150 ml, bid) + E	D	14	(1)
Yao 2014 [24]	203/189	25–78/26–79	Pelvic tumor	NR	NOHS (2002)	Re + Po	mBTWD (50 ml, qd, Re) + (100 ml, bid, Po)	Antibiotic (iv) + A + B (50 ml, qd)	20	(1), (3), (7)
Li 2008 [20]	32/27	32–65	Abdominal tumor	NR	GGCCDT (2003)	Re	mBTWD (100 ml, qd) + Symptomatic treatment	Symptomatic treatment	15	(1)
Tong 2012 [25]	47/42	56/53	Pelvic tumor	NRT	MOR (2001)	Re + Po	mBTWD (50 ml, qd)	C (50 ml, qd)	NR	(3), (4), (7)
Ma 2021 [9]	30/30	57.87 ± 6.62/58.83 ± 5.97	Abdominal tumor	NRT	CCDTRP (2018)	Re	BTWD (100 ml, qd)	C (100 ml, qd)	20	(1), (2), (3), (5), (6)
Wang 2017 [21]	42/42	60.5 ± 2.1/63.0 ± 3.2	NR	NR	NR	Re	mBTWD (200 ml, qd) + Xilei San (3 g)	B + adrenalin (200 ml, qd)	NR	(1), (2)
Zhang 2013 [22]	34/34	56.62 ± 9.82/52.29 ± 9.77	NR	NR	CCDTRP (2018)	Re	mBTWD (200 ml, qd) + Xilei San (3 g)	B + adrenalin (200 ml, qd)	14	(1), (2)
Ye 2007 [28]	43/43	NR	Pelvic tumor	NR	NR	Po	mBTWD	Blank control	NR	(1)
Wang 2021 [29]	27/34	52.7 ± 5.6/51.3 ± 4.4	Pelvic tumor	NRT	CCDTRP (2018)	Po	mBTWD (200 ml, bid) + Montmorillonite (3 g, tid)	Montmorillonite (3 g, tid)	NR	(1), (2), (3), (4)

Note. NR, not report. RNT, random number table. CCDTRP (2018), the consensus of Chinese experts on diagnosis and treatment of radiation proctitis (2018). NOHS (2002), national occupational health standards (2002). MOR (2001), modern oncology radiation therapy (2001). SDTMTC, standard for the diagnosis and treatment of common malignant tumors in China. GGCCDT (2003), graphic gastroenterology -clinical classical diagnosis and treatment methods (2003). Re, retention enema. Po, per os. BTWD, Baitouweng decoction. mBTWD, modified BTWD. A, 0.9% NaCl solution 50 ml + gentamicin 160,000 U + dexamethasone 10 mg + montmorillonite powder 3 g. B, 0.9% NaCl solution 100 ml + gentamicin 160,000 U + dexamethasone 5 mg + procaine/lidocaine 10 ml. C, 0.9% NaCl solution 100 ml + dexamethasone 10 mg + montmorillonite powder 6 g. D, norfloxacin (0.2 g, tid) + berberine (0.3 g, tid) + yunnan baiyao (0.5 g, tid). rhGM-CSF, recombinant human granulocyte-macrophagecolony-stimulating factor for injection. (1) clinical efficacy, (2) TCM symptom score, (3) colonoscopy score (grade) and radiation enteritis grading, (4) KPS score, (5) serum inflammatory factor level, (6) fecal occult blood, and (7) adverse events and recurrence rate.

**Table 2:** mBTWD composition of included studies.

First-author (year)	Formula	Components
Chen 2020 [14]	mBTWD	Pulsatillia Radix (Baitouweng, Chinese bulbul) 12 g; Coptidis Rhizoma (Huanglian, Goldthread) 12 g; Phellodendri Chinrnsis Cortex (Huangbo, Tractat) 12 g; Fraxini Cortex (Qinpi, Ash bark) 15 g; Burnet (Diyu, Sanguisorba officinalis) 15 g; Saposhnicovia divaricate (Fangfeng, Radix sileris) 9 g

Zhao 2011 [26]	mBTWD	Pulsatilliae Radix (Baitouweng, Chinese bulbul) 15 g; Coptidis Rhizoma (Huanglian, Goldthread) 5 g; Phellodendri Chinrnsis Cortex (Huangbo, Tractat) 10 g; Fraxini Cortex (Qinpi, Ash bark) 10 g; Sophora japonica (Huaihua, Flos sophorae) 10 g; Aucklandia (Muxiang, Costustoot) 10 g; Pueraria (Gegen, The root of kudzu vine) 15 g; Corydalis tuber (Yanhusuo, Rhizoma corydalis) 10 g; Licorice (Gancao, Glycyrrhiza) 6 g; Atractylodes (Baizhu, Rhizoma atractylodis) 10 g; Nepeta (Jingjie, Schizonepeta) 10 g; Red peony root (Chishao, Radix paeoniae rubra) 10 g; Rheum officinale (Dahuang, Chinese rhubarb) 6 g; Coloured malt (Maiya, Malt culms) 10 g; Amomum (Sharen, Fructus amomi) 3 g

Jiang 2008 [23]	mBTWD	Pulsatilliae Radix (Baitouweng, Chinese bulbul) 15 g; Coptidis Rhizoma (Huanglian, Goldthread) 6 g; Phellodendri Chinrnsis Cortex (Huangbo, Tractat) 10 g; Burnet (Diyu, Sanguisorba officinalis) 15 g; Red peony root (Chishao, Radix paeoniae rubra) 30 g; White peony root (Baishao, Radix paeoniae alba) 15 g; Angelica sinensis (Danggui, Chinese angelica) 20 g; Paeonia suffruticosa (Mudanpi, Moutan bark) 15 g; Radix scrophulariae (Xuanshen, Figwort root) 15 g; Field thistle (Xiaoji, cephalanoplos segetum) 10 g; Notoginseng powder (Sanqifen, Notoginseng root) 3 g; Cacumen biotae (Cebaiye, Chinese arborvitae twig) 15 g; Sophora japonica (Huaihua, Flos sophorae) 15 g; Licorice (Gancao, Glycyrrhiza) 10 g

Lai 2021 [15]	mBTWD	Pulsatilliae Radix (Baitouweng, Chinese bulbul) 30 g; Coptidis Rhizoma (Huanglian, Goldthread) 30 g; Radix Scutellariae (Huangqin, Scutellaria baicalensis) 30 g; Burnet (Diyu, Sanguisorba officinalis) 15 g; Sophora japonica (Huaihua, Flos sophorae) 15 g; Pueraria (Gegen, The root of kudzu vine) 30 g; Cultured calculus bovis (Tiwaipeiyuniuhuang, Cultured bezoar in vitro) 0.3 g; Philippine violet herb (Zihuadiding, Chinese violet) 15 g

Lei 2019 [16]	mBTWD	Pulsatilliae Radix (Baitouweng, Chinese bulbul) 15 g; Coptidis Rhizoma (Huanglian, Goldthread) 10 g; Phellodendri Chinrnsis Cortex (Huangbo, Tractat) 10 g; Fraxini Cortex (Qinpi, Ash bark) 15 g; Burnet (Diyu, Sanguisorba officinalis) 15 g; White peony root (Baishao, Radix paeoniae alba) 15 g; Angelica sinensis (Danggui, Chinese angelica) 15 g; Poria cocos (Fuling, Tuckahoe) 15 g; Rheum officinale (Dahuang, Chinese rhubarb) 5 g; Aucklandia (Muxiang, Costustoot) 15 g; Areca-nut (Binglang, Areca catechu) 15 g; Rhizoma atractylodis (Cangzhu, Atractylodes) 15 g; Licorice (Gancao, Glycyrrhiza) 6 g

Lei 2010 [17]	mBTWD	Pulsatilliae Radix (Baitouweng, Chinese bulbul) 10 g; Coptidis Rhizoma (Huanglian, Goldthread) 5 g; Phellodendri Chinrnsis Cortex (Huangbo, Tractat) 5 g; Fraxini Cortex (Qinpi, Ash bark) 10 g; Burnet (Diyu, Sanguisorba officinalis) 15 g; Sophora flavescens (Kushen, Radix sophorae flavescentis) 10 g; Common bletilla pseudobulb (Baiji, Rhizoma bletillae) 10 g; Aucklandia (Muxiang, Costustoot) 10 g; Hedyotis diffusa (Baihuasheshecao, Oldenlandia) 10 g; Corydalis tuber (Yanhusuo, Rhizoma corydalis) 10 g; Red peony root (Chishao, Radix paeoniae rubra) 10 g

Xia 2016 [18]	mBTWD	Pulsatilliae Radix (Baitouweng, Chinese bulbul) 30 g; Coptidis Rhizoma (Huanglian, Goldthread) 30 g; Phellodendri Chinrnsis Cortex (Huangbo, Tractat) 30 g; Sophora flavescens (Kushen, Radix sophorae flavescentis) 30 g; Common bletilla pseudobulb (Baiji, Rhizoma bletillae) 30 g; Hedyotis diffusa (Baihuasheshecao, Oldenlandia) 30 g; White peony root (Baishao, Radix paeoniae alba) 30 g

Yang 2012 [19]	mBTWD	Pulsatilliae Radix (Baitouweng, Chinese bulbul) 30 g; Coptidis Rhizoma (Huanglian, Goldthread) 30 g; Phellodendri Chinrnsis Cortex (Huangbo, Tractat) 30 g; Burnet (Diyu, Sanguisorba officinalis) 30 g; Sophora japonica (Huaihua, Flos sophorae) 30 g; Porslane (Machixian, Portulacae herba) 30 g; Honeysuckle (Jinyinhua, Lonicera japonica) 30 g

Jiang 2011 [27]	mBTWD	Pulsatilliae Radix (Baitouweng, Chinese bulbul) 15 g; Coptidis Rhizoma (Huanglian, Goldthread) 3 g; Phellodendri Chinrnsis Cortex (Huangbo, Tractat) 6 g; Fraxini Cortex (Qinpi, Ash bark) 9 g; Burnet (Diyu, Sanguisorba officinalis) 12 g; Dried rehmannia root (Shengdihuang, Radix rehmanniae recen) 12 g; Paeonia suffruticosa (Mudanpi, Moutan bark) 6 g; Red peony root (Chishao, Radix paeoniae rubra) 9 g; Sophora japonica (Huaihua, Flos sophorae) 12 g; Licorice (Gancao, Glycyrrhiza) 6 g

Yao 2014 [24]	mBTWD	Pulsatilliae Radix (Baitouweng, Chinese bulbul) 15 g; Coptidis Rhizoma (Huanglian, Goldthread) 6 g; Fraxini Cortex (Qinpi, Ash bark) 10 g; Burnet (Diyu, Sanguisorba officinalis) 20 g; Astragalus membranaceus (Huangqi, Radix Astragali) 30 g; Atractylodes (Baizhu, Rhizoma atractylodis) 10 g; Angelica sinensis (Danggui, Chinese angelica) 10 g; Dried rehmannia root (Shengdihuang, Radix rehmanniae recen) 15 g; White peony root (Baishao, Radix paeoniae alba) 15 g; Taraxacum (Pugongying, Dandelion) 10 g; Aucklandia (Muxiang, Costustoot) 6 g; Areca-nut (Binglang, Areca catechu) 6 g; Licorice (Gancao, Glycyrrhiza) 5 g

Li 2008 [20]	mBTWD	Pulsatilliae Radix (Baitouweng, Chinese bulbul) 15 g; Coptidis Rhizoma (Huanglian, Goldthread) 6 g; Phellodendri Chinrnsis Cortex (Huangbo, Tractat) 12 g; Fraxini Cortex (Qinpi, Ash bark) 12 g; Burnet (Diyu, Sanguisorba officinalis) 15 g; Saposhnicovia divaricata (Fangfeng, Radix sileris) 12 g

Tong 2012 [25]	mBTWD	Pulsatilliae Radix (Baitouweng, Chinese bulbul) 15 g; Coptidis Rhizoma (Huanglian, Goldthread) 12 g; Radix Scutellariae (Huangqin, Scutellaria baicalensis) 6 g; Fraxini Cortex (Qinpi, Ash bark) 12 g; Pueraria (Gegen, The root of the kudzu vine) 9 g; Fructus schizandrae (Wuweizi, Chinese magnoliavine fruit) 6 g; Cuttle bone (Wuzeigu, The inkfish bone) 10 g

Ma 2021 [9]	BTWD	Pulsatilliae Radix (Baitouweng, Chinese bulbul) 30 g; Coptidis Rhizoma (Huanglian, Goldthread) 12 g; Phellodendri Chinrnsis Cortex (Huangbo, Tractat) 24 g; Fraxini Cortex (Qinpi, Ash bark) 24 g

Wang 2017 [21]	mBTWD	Pulsatilliae Radix (Baitouweng, Chinese bulbul) 30 g; Coptidis Rhizoma (Huanglian, Goldthread) 10 g; Phellodendri Chinrnsis Cortex (Huangbo, Tractat) 10 g; Fraxini Cortex (Qinpi, Ash bark) 20 g; Burnet (Diyu, Sanguisorba officinalis) 10 g; White peony root (Baishao, Radix paeoniae alba) 10 g; Red peony root (Chishao, Radix paeoniae rubra) 10 g; Sophora japonica (Huaihua, Flos sophorae) 10 g; Patrinia (Baijiangcao, White flower Patrinia Herb) 30 g; Aucklandia (Muxiang, Costustoot) 6 g

Zhang 2013 [22]	mBTWD	Pulsatilliae Radix (Baitouweng, Chinese bulbul) 30 g; Coptidis Rhizoma (Huanglian, Goldthread) 10 g; Phellodendri Chinrnsis Cortex (Huangbo, Tractat) 10 g; Fraxini Cortex (Qinpi, Ash bark) 20 g; Burnet (Diyu, Sanguisorba officinalis) 10 g; White peony root (Baishao, Radix paeoniae alba) 10 g; Red peony root (Chishao, Radix paeoniae rubra) 10 g; Sophora japonica (Huaihua, Flos sophorae) 10 g; Common bletilla pseudobulb (Baiji, Rhizoma bletillae) 10 g; Patrinia (Baijiangcao, White flower Patrinia Herb) 30 g; Aucklandia (Muxiang, Costustoot) 6 g

Ye 2007 [28]	mBTWD	Pulsatilliae Radix (Baitouweng, Chinese bulbul) 10 g; Coptidis Rhizoma (Huanglian, Goldthread) 12 g; Phellodendri Chinrnsis Cortex (Huangbo, Tractat) 12 g; Fraxini Cortex (Qinpi, Ash bark) 12 g; Burnet (Diyu, Sanguisorba officinalis) 15 g; Porslane (Machixian, Portulacae herba) 30 g; Sophora flavescens (Kushen, Radix sophorae flavescentis) 10 g; Sophora japonica (Huaihua, Flos sophorae) 15 g; Coix Seed (Yiyiren, Semen coicis) 20 g; Aucklandia (Muxiang, Costustoot) 10 g

Wang 2021 [29]	mBTWD	Pulsatilliae Radix (Baitouweng, Chinese bulbul) 15 g; Phellodendri Chinrnsis Cortex (Huangbo, Tractat) 10 g; Radix Scutellariae (Huangqin, Scutellaria baicalensis) 10 g; Red peony root (Chishao, Radix paeoniae rubra) 20 g; Angelica sinensis (Danggui, Chinese angelica) 15 g; Aucklandia (Muxiang, Costustoot) 10 g; Patrinia (Baijiangcao, White flower Patrinia Herb) 10 g; Coix Seed (Yiyiren, Semen coicis) 10 g; White peony root (Baishao, Radix paeoniae alba) 10 g; Citrus (Chenpi, Tangerine Peel) 10 g; Hairyvein agrimony (Xianhecao, Agrimonia pilosa ledeb) 10 g; Licorice (Gancao, Glycyrrhiza) 5 g 1.Hemafecia: Sophora japonica (Huaihua, Flos sophorae) 10 g, Burnet (Diyu, Sanguisorba officinalis) 15 g, Palm shell charcoal (Zongyutan, Palm kernel shell activated carbon) 15 g

Note. mBTWD, modified Baitouweng decoction.

**Table 3:** Univariate meta-regression analyses of clinical efficacy.

Univariate meta-regressionmodels	No. of studies	*I* ^2^ (%)	Tau^2^ (%)	*P* value	Estimated risk ratio^*∗*^ (95% CI)
*Intervention*					
mBTWD with CWM	6	39.730	0.328	0.852	1.014 (0.859, 1.198)
mBTWD without CWM	9				

*Drug deliver*					
Re	9	38.560	0.321	0.800	1.030 (0.801, 1.326)
Po	4			0.520	0.941 (0.770, 1.149)
Re + Po	2				
Sample size	15	39.440	0.004	0.878	1.000 (0.993, 1.001)
Mean age	14^▲^	34.660	0.003	0.747	1.002 (0.984, 1.022)
Duration	12^■^	45.510	0.006	0.849	1.000 (0.994, 1.007)
Publication year	15	38.400	0.003	0.567	0.995 (0.978, 1.013)

Note. ^*∗*^Statistics including Tau^2^, *I*^2^, and *P* values are derived from meta-regression models conducted with Stata 14.0, estimated risk ratio is obtained using the conventional fixed-effects model with 95% confidence interval (CI). Both dichotomous and continuous covariates are employed in the regression models. ^▲^Ye and Wang [28] report related data without mean age. ^■^Ye and Wang [28], Wang [21], and Wang et al. [29] report related data without duration time.

**Table 4:** GRADE evidence for mBTWD in treating radiation enteritis.

No. of studies	Study design	Risk of bias	Inconsistency	Indirectness	Imprecision	Other considerations	Quality of evidence
*Clinical efficacy*							
15	RCTs	Serious	Serious	Not serious	Not serious	Publication bias	⊕○○○ very low

*Tcm syndrome total score*							
6	RCTs	Serious	Not serious	Not serious	Not serious	None	⊕⊕⊕○ moderate

*Colonoscopy score*							
2	RCTs	Serious	Not serious	Not serious	Not serious	None	⊕⊕⊕○ moderate

*Colonoscopy grade (0*–*II)*							
2	RCTs	Serious	Not serious	Not serious	Not serious	None	⊕⊕⊕○ moderate

*Colonoscopy grade (III-IV)*							
2	RCTs	Serious	Not serious	Not serious	Not serious	None	⊕⊕⊕○ moderate

*Radiation enteritis grading (0*–*II)*							
6	RCTs	Serious	Not serious	Not serious	Not serious	None	⊕⊕⊕○ moderate

*Radiation enteritis grading (III-IV)*							
6	RCTs	Serious	Not serious	Not serious	Not serious	None	⊕⊕⊕○ moderate

*CRP*							
2	RCTs	Serious	Not serious	Not serious	Not serious	None	⊕⊕⊕○ moderate

*KPS*							
3	RCTs	Serious	Serious	Not serious	Not serious	None	⊕⊕○○ low

*OB*							
2	RCTs	Serious	Not serious	Not serious	Not serious	None	⊕⊕⊕○ moderate

*Recurrence rate*							
2	RCTs	Serious	Serious	Not serious	Serious	None	⊕○○○ very low

Moderate quality means the estimated effect sizes and their confidence levels may be affected by further studies; low quality represents that the estimated effect sizes and their confidence levels are likely to be affected or changed by further research; very low-quality shows that the estimated effect sizes and their confidence levels are confronted of huge uncertainty.

**Table 5:** The main active ingredients of representative drugs in mBTWD.

Mol ID	MolName	OBA (%)	DL	Herb
MOL001971	Pulchinenoside c_qt	37.79	0.76	BTW
MOL001973	Sitosteryl acetate	40.39	0.85	BTW
MOL001978	Aureusidin	53.42	0.24	BTW
MOL001979	Lan	42.12	0.75	BTW
MOL001984	3beta, 23-Dihydroxy-lup-20 (29)-ene-28-o-alpha-l-rhamnopyranosyl-(1-4)-beta-d-glucopyranosyl (1-6)-beta-d-glucopyranoside_qt	37.59	0.79	BTW
MOL001985	Zinc01615307	56.38	0.87	BTW
MOL001987	Β-sitosterol	33.94	0.7	BTW
MOL000211	Mairin	55.38	0.78	BTW
MOL000354	Isorhamnetin	49.6	0.31	BTW
MOL000449	Stigmasterol	43.83	0.76	BTW, HB
MOL000358	Beta-sitosterol	36.91	0.75	BTW, HB, QP
MOL001454	Berberine	36.86	0.78	HB, HL
MOL001458	Coptisine	30.67	0.86	HB, HL
MOL002636	Kihadalactone a	34.21	0.82	HB
MOL013352	Obacunone	43.29	0.77	HB, HL
MOL002641	Phellavin_qt	35.86	0.44	HB
MOL002643	Delta 7-stigmastenol	37.42	0.75	HB
MOL002644	Phellopterin	40.19	0.28	HB
MOL002651	Dehydrotanshinone ii a	43.76	0.4	HB
MOL002652	Delta7-dehydrosophoramine	54.45	0.25	HB
MOL002656	Dihydroniloticin	36.43	0.81	HB
MOL002659	Kihadanin a	31.6	0.7	HB
MOL002660	Niloticin	41.41	0.82	HB
MOL002662	Rutaecarpine	40.3	0.6	HB, HL
MOL002663	Skimmianin	40.14	0.2	HB
MOL002666	Chelerythrine	34.18	0.78	HB
MOL002668	Worenine	45.83	0.87	HB, HL
MOL002670	Cavidine	35.64	0.81	HB
MOL002671	Candletoxin a	31.81	0.69	HB
MOL002672	Hericenone h	39	0.63	HB
MOL002673	Hispidone	36.18	0.83	HB
MOL000622	Magnograndiolide	63.71	0.19	HB, HL
MOL000762	Palmidin a	35.36	0.65	HB, HL
MOL000785	Palmatine	64.6	0.65	HB, HL
MOL000787	Fumarine	59.26	0.83	HB
MOL000790	Isocorypalmine	35.77	0.59	HB
MOL000098	Quercetin	46.43	0.28	HB, HL
MOL001131	Phellamurin_qt	56.6	0.39	HB
MOL001455	(s)-canadine	53.83	0.77	HB
MOL001771	Poriferast-5-en-3beta-ol	36.91	0.75	HB
MOL002894	Berberrubine	35.74	0.73	HB, HL
MOL005438	Campesterol	37.58	0.71	HB
MOL006401	Melianone	40.53	0.78	HB
MOL006413	Phellochin	35.41	0.82	HB
MOL006422	Thalifendine	44.41	0.73	HB
MOL002897	Epiberberine	43.09	0.78	HL
MOL002903	(r)-canadine	55.37	0.77	HL
MOL002904	Berlambine	36.68	0.82	HL
MOL002907	Corchoroside a_qt	104.95	0.78	HL
MOL006709	Aids214634	92.43	0.55	QP
MOL006710	8-(beta-d-glucopyranosyloxy)-7-hydroxy-6-methoxy-2h-1-benzopyran-2-one	36.76	0.42	QP

**Table 6:** Molecular docking of active ingredients with key targets from representative drugs in mBTWD.

Target protein	PDB ID	PubChem CID	MolName	Affinity/kJ·mol^−1^
MYC	1A93	5743	Dexamethasone	−0.0
		5280343	Quercetin	−2.8
TP53	6WQX	5743	Dexamethasone	−7.9
		5280343	Quercetin	−8.3

MAPK14	1A9U	5743	Dexamethasone	−5.4
		5281220	Aureusidin	−7.7
		5281654	Isorhamnetin	−7.2

MAPK1	1PME	5743	Dexamethasone	−5.5
		5280343	Quercetin	−8.5

## Abbreviations

CNKI: China national knowledge infrastructure
CWM: Conventional western medicine
DL: Drug like
GO: Gene ontology
GRADE: Grading of recommendations assessment, development and evaluation
INPLASY: International platform of registered systematic review and meta-analysis protocols
KEGG: Kyoto encyclopedia of genes and genomes
mBTWD: Modified baitouweng decoction
NR: Not reported
OB: Occult blood
OBA: Oral bioavailability
OR: Odds ratio
Po: Per os
RCTs: Randomised controlled trials
Re: Reserved enema
rhGM-CSF: Recombinant human granulocyte-macrophage colony stimulating factor for injection
RNT: Random number table
ROS: Reactive oxide species
RR: Risk ratio
TCM: Traditional chinese medicine
TCMSP: Traditional chinese medicine systems pharmacology.
